# Reading between the troponin lines: acute myocarditis amid septic shock

**DOI:** 10.1093/ehjcr/ytag386

**Published:** 2026-05-22

**Authors:** Tai Meng Chen, Sandeep Singh Hothi, Timothy C Tan, Clement Lau

**Affiliations:** Department of Cardiology, National Heart Centre Singapore, Singapore 169609, Singapore; Department of Cardiology, Hospital Raja Permaisuri Bainun, Ipoh 30450, Malaysia; Heart and Lung Centre, Royal Wolverhampton NHS Trust, Wolverhampton, WV10 0QP, UK; Institute of Cardiovascular Sciences, University of Birmingham, Birmingham, B15 2TT, UK; Department of Cardiology, Blacktown Hospital, Blacktown, NSW 2148, Australia; School of Medicine, Western Sydney University, Sydney, NSW 2751, Australia; Department of Cardiology, National Heart Centre Singapore, Singapore 169609, Singapore

**This editorial refers to ‘Beyond the gut: a case report of Shigella-associated myocarditis’, by L.C. Maher *et al.*  https://doi.org/10.1093/ehjcr/ytag171.**

Acute myocarditis (AM) is increasingly encountered in acute cardiac services, driven by improved detection with high-sensitivity troponin assays and wider availability of cardiac magnetic resonance (CMR). Distinguishing ‘true’ myocarditis from septic cardiomyopathy, acute coronary syndrome (ACS), and myocardial infarction with non-obstructive coronary arteries (MINOCA) remains a key clinical challenge with important implications for prognosis, management, and patient counselling.

Maher and colleagues reported a striking case of severe shigellosis with multi-organ failure and a rare cardiac manifestation consistent with AM.^1^ Acute chest pain, dynamic ECG changes, and elevated troponin suggested myocardial infarction. After CT coronary angiography (CTCA) excluded obstructive disease, attention shifted to alternative causes of myocardial injury. CMR proved decisive, providing tissue characterization consistent with AM and allowing confident distinction from infarction and sepsis-related myocardial dysfunction.

This case highlights that AM remains an important differential whenever troponin elevation and ECG changes occur during systemic inflammation or sepsis. It also illustrates the need to adapt guidelines to haemodynamic instability and competing diagnoses. *Figure 1* outlines a practical diagnostic approach, reflecting recommendations in the 2025 ESC guidelines for myocarditis and pericarditis.

**Figure 1:**
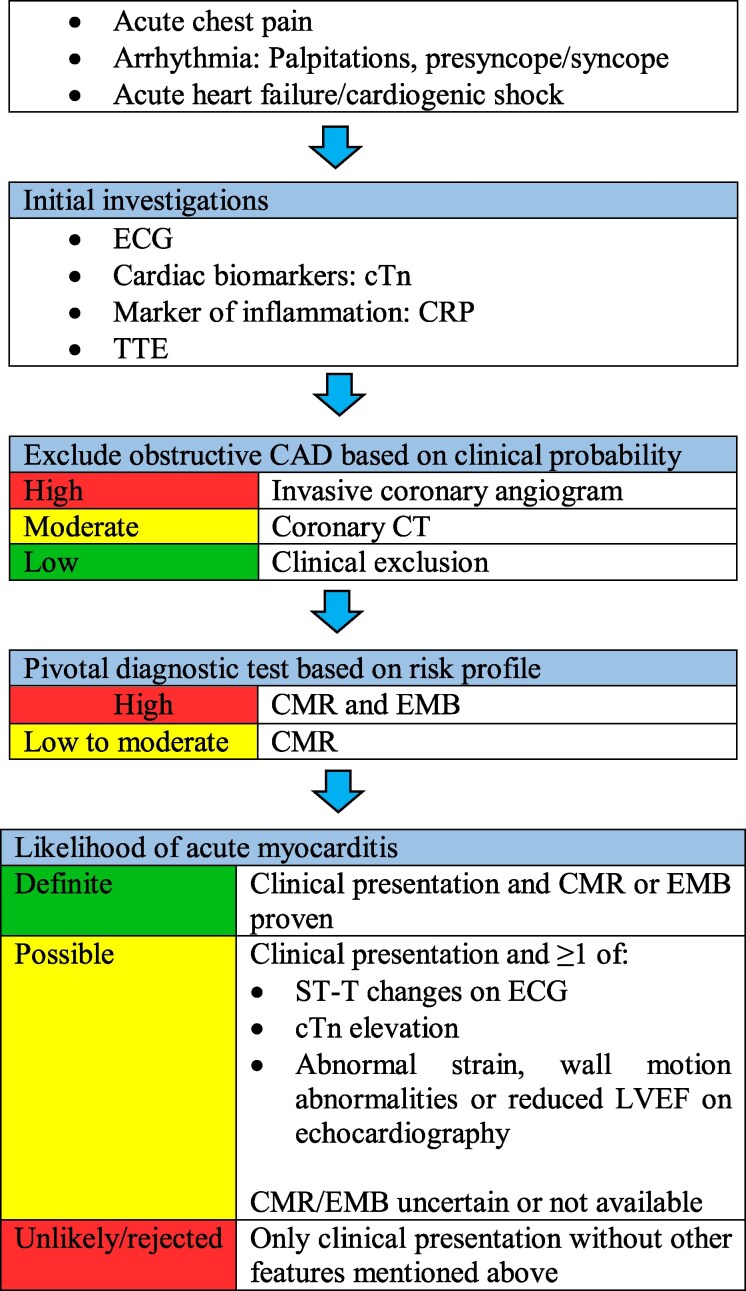
A simplified diagnostic flowchart for acute myocarditis. CAD, coronary artery disease; CMR, cardiovascular magnetic resonance; CRP, C-reactive protein; cTn, cardiac troponin; ECG, electrocardiography; EMB, endomyocardial biopsy; LVEF, left ventricular ejection fraction; TTE, transthoracic echocardiography.

## When to suspect acute myocarditis

Myocarditis is an inflammatory myocardial disorder, usually triggered by viral infection, toxins, or systemic immune activation.^2,3^ The clinical spectrum ranges from asymptomatic episodes to fulminant presentations with cardiogenic shock and lethal arrhythmias. AM is defined by a symptom-to-diagnosis interval of less than one month, with subacute and chronic forms reflecting more protracted courses.^3^ Fulminant myocarditis represents the extreme of this spectrum, and carries a substantial early mortality despite a favourable long-term outlook for survivors.^4^

## Clinical presentation

Three classical presentations should raise the possibility of AM: an ACS-like picture with chest pain and troponin rise; new-onset arrhythmia, including tachyarrhythmias or high-grade atrioventricular block; and acute heart failure, which may progress to cardiogenic shock. Registry data identify chest pain as the most frequent symptom (>85%), followed by dyspnoea (19–49%), while palpitations and syncope are less frequent.^5–9^ A febrile or ‘viral’ prodrome, often respiratory or gastrointestinal, precedes the acute phase and should prompt consideration of AM in younger individuals with otherwise unexplained myocardial injury.^5–7,9^

## Bedside investigations: what they can and cannot tell us

The ECG is abnormal in approximately 85% of AM patients. Recognized abnormalities include ST-T-wave changes and QRS widening. While no pattern is pathognomonic, QRS >120 ms, sustained ventricular tachyarrhythmias, and high-grade atrioventricular block (AVB) suggest a complicated course.^10^ High-grade AVB in patients with preserved left ventricular ejection fraction (LVEF) >50% should prompt consideration of cardiac sarcoidosis, Lyme carditis, or immune-checkpoint-inhibitor-associated myocarditis.^11^

Cardiac troponin is usually elevated, yet it correlates poorly with left ventricular systolic dysfunction (LVSD) severity and cannot discriminate between myocarditis, myocardial infarction, or sepsis-related injury.^12,13^ Inflammatory markers (C-reactive protein) are frequently raised but non-specific. In septic shock cases like Maher’s, troponin rise and LVSD may simply reflect systemic illness unless corroborated by imaging or histology.

Transthoracic echocardiography is the first-line imaging modality in AM. Beyond LVEF, assessment may reveal wall thickening from interstitial oedema, regional wall motion abnormalities (often inferior or inferolateral walls), diastolic dysfunction, or small pericardial effusions. LVEF at presentation is a strong prognostic marker.^4,5,7^ Strain imaging, particularly global longitudinal strain, enhances sensitivity even with preserved LVEF.^14^

Given the overlap between AM and ACS, particularly in younger patients with chest pain, ECG changes, and troponin elevation, excluding obstructive coronary artery disease is essential. The choice between invasive angiography and CTCA should be guided by pre-test probability, haemodynamic status, and local expertise.

## Cardiovascular magnetic resonance and modern diagnostic paradigm

CMR is central to myocarditis diagnosis and phenotyping, integrating functional assessment, tissue characterization, and pericardial evaluation in one examination. T2-based techniques (T2-weighted imaging and mapping) detect myocardial oedema, while T1 mapping and extracellular volume quantify inflammation and diffuse fibrosis.^15,16^ Late gadolinium enhancement (LGE) identifies focal necrosis and replacement fibrosis, typically in non-ischaemic patterns (mid-wall, or sub-epicardial), often in lateral or inferior walls.^15^

The updated 2018 Lake Louise Criteria define active inflammation when at least one T2-based or one T1-based criterion is present. LVSD and pericardial changes are supportive features.^15^ The diagnostic yield of CMR is time-dependent, peaking (56–81% of T2-weighted CMR abnormalities) within 1–2 weeks of symptom onset and decreasing as inflammation resolves. Conversely, LGE may persist and provide longer-term evidence of prior injury.^17^ The 2025 ESC guidelines place CMR at the core of diagnostic work-up, recommending follow-up CMR within six months to assess resolution or identify persistent inflammation and scar.^3^ Where CMR is unfeasible or inconclusive, FDG-PET offers a complementary role, especially for suspected cardiac sarcoidosis.^3^

In Maher’s case, haemodynamic instability and multi-organ failure delayed CMR until the patient had stabilized. In critically ill patients, the priority remains resuscitation and controlling the systemic insult with CMR done only after stabilization of the patient. This case exemplifies how the ESC framework requires clinical judgement rather than dogmatic adherence.

## Endomyocardial biopsy: who, why, and when?

Endomyocardial biopsy (EMB) remains the reference standard for definitive diagnosis in selected patients, allowing histological sub-classification, detection of viral genomes, and identification of unexpected pathology.^2,18^ Contemporary ESC guidance supports EMB (Class I) in high-risk presentations, that is, fulminant myocarditis, refractory heart failure, malignant arrhythmias, or high-grade atrioventricular block, and in intermediate-risk patients who fail therapy.^3^ Potential complications, including cardiac perforation, tamponade, thromboembolism, and severe arrhythmias, are rare in experienced hands but not negligible.^18–20^ In septic shock and multi-organ failure, the risk–benefit balance is particularly delicate. In Maher’s case, the clear infectious trigger, typical CMR findings, and clinical improvement under antimicrobial therapy rendered biopsy unnecessary.

## Guideline strengths and gaps highlighted by this case

The 2025 ESC guidelines on myocarditis and pericarditis offer unified terminology and presentation-driven algorithms integrating ECG, biomarkers, multi-modality imaging, and EMB. However, the Maher case highlights evidential gaps. First, the guidelines provide limited information on rare bacterial causes like *Shigella*. Second, in septic shock, features that define ‘possible’ or ‘definite’ myocarditis, that is, tachycardia, troponin elevation, LVSD, may be driven by sepsis itself, and CMR or biopsy can help in this situation. Third, while endorsing early CMR, the guideline lacks precise timing for haemodynamically unstable patients. This case illustrates a pragmatic ‘resuscitate first, image later’ approach, consistent with good practice but not explicitly captured in algorithms. Finally, EMB indications in severe bacterial sepsis remain under-defined, requiring clinicians to extrapolate from viral myocarditis data.

## Conclusion

Robust imaging and 2025 ESC guidelines support structured, evidence-based diagnosis.^3^ Maher’s case reminds us that in severe systemic infection, troponin elevation, and LVSD cannot be taken at face value, and careful application, rather than rigid adherence to guidelines, is required. It also underscores the need for prospective data from under-represented populations, including bacterial sepsis-associated myocarditis, to inform future ESC recommendations.

## Data Availability

No new data were generated or analysed in support of this editorial.

## Lead author biography

Dr. Tai Meng Chen is a cardiology fellow from Hospital Raja Permaisuri Bainun, Ipoh, Malaysia. He is currently undergoing advanced training in echocardiography at National Heart Centre Singapore. He has a special interest in transthoracic and transoesophageal echocardiography, cardiac MRI, cardiac CT, and coronary interventions.
